# Development of simultaneous interaction prediction approach (SiPA) for the expansion of interaction network of traditional Chinese medicine

**DOI:** 10.1186/s13020-020-00369-z

**Published:** 2020-08-26

**Authors:** Mengjie Rui, Hui Pang, Wei Ji, Siqi Wang, Xuefei Yu, Lilong Wang, Chunlai Feng

**Affiliations:** School of Pharmacy, Jiangsu University, Zhenjiang, 212013 People’s Republic of China

**Keywords:** Traditional Chinese medicine, Network pharmacology, Interaction prediction, Simple inference model, Compound-target correlation space based interaction prediction model, Canonical correlation analysis

## Abstract

**Background:**

Due to the lack of enough interaction data among compositions, targets and diseases, it is difficult to construct a complete network of Traditional Chinese Medicine (TCM) that comprehensively reflects active compositions and their synergistic network in terms of specific diseases. Therefore, mapping of the full spectrum of interaction between compounds and their targets is of central importance when we use network pharmacology approach to explore the therapeutic potential of the TCM.

**Methods:**

To address this challenge, we developed a large-scale simultaneous interaction prediction approach (SiPA) integrated one interaction network based simple inference model (SIM), focusing on ‘logical relevance’ between compounds, proteins or diseases, and another compound-target correlation space based interaction prediction model (CTCS-IPM) that was built on the basis of the canonical correlation analysis (CCA) to estimate the position of compounds (or targets) in compound-protein correlated space. Then SiPA was applied to discover reliable multiple interactions for interaction network expansion of a TCM, compound *Salvia miltiorrhiza*. By means of network analysis, potential active compounds and their related network synergy underlying cardiovascular diseases were evaluated between expanded and original interaction networks. Part of new interactions were validated with existing experimental evidence and molecular docking.

**Results:**

As evaluated with known test dataset, the established combination approach was proved to make highly accurate prediction, showing a well prediction performance for the SIM and a high recall rate of 85.2% for the CTCS-IPM. Then 710 pairs of new compound-target interactions, 24 pairs of new compound-cardiovascular disease interactions and 294 pairs of new cardiovascular disease-protein interactions were predicted for compound *Salvia miltiorrhiza*. Results of network analysis suggested the network expansion could dramatically improve the completeness and effectiveness of the network. Validation results of literature and molecular docking manifested that inferred interactions had good reliability.

**Conclusions:**

We provided a practical and efficient way for large-scale inference of multiple interactions of TCM ingredients, which was not limited by the lack of negative samples, sample size and target 3D structures. SiPA could help researchers more accurately prioritize the effective compounds and more completely explore network synergy of TCM for treating specific diseases, indicating a potential way for effectively identifying candidate compound (or target) in drug discovery.

## Background

Traditional Chinese Medicine (TCM) has held, and continues to occupy, an important position in health care within China and other East Asian countries, and has increasingly aroused broad attention in scientific communities throughout the world. The revival of interest in TCM partly stems from the hope that TCM can act in a synergistic manner to improve therapeutic efficacy, because of its “Multi-components and multi-targets” property. However, the complicated compositions of the TCM have posed a great challenge to identify the active combinations of chemical constituents and to prove their mechanism of actions [1]. To address this issue, network pharmacology approach currently provides an alternative way to systematically investigate therapeutic effects of dozens of constitutes in TCM [2–6]. In practice, this concept has been regarded with many skepticism by the fact that a comprehensive pharmacointeraction network of TCM is constructed, in many cases, with great difficulty, as a result of the insufficient information about all the possible composition-target interactions in one TCM prescription [7, 8]. Therefore, mapping of the full spectrum of interaction between compounds and their targets is of central importance when we use network pharmacology approach to explore the therapeutic potential of the TCM.

Considering that numerous chemical compositions, and diverse cellular targets are involved in the synergistic or antagonistic effects of TCM, the trial-and-error experimental approaches are rather time- and money-consuming to identify novel composition-target interactions. Recently, computational approaches like chemical similarity search [9], pharmacophore model [10], reverse molecular docking [11], machine learning [12] and combination of multiple approaches [13] were developed for the inference of interactions between compositions and targets. Meanwhile, various online tools have been developed to provide valuable supports for identifying potential targets of compounds, for example, Similarity Ensemble Approach (SEA), identifing targets based on chemical 2D similarity [14]; ChemMapper, predicting targets and mode of action for small molecules based on 3D similarity computation [15]; PharmMapper, a Pharmacophore model based prediction [16]; TarfisDock, using reverse ligand–protein docking to seek potential protein targets by screening an appropriate protein database [17]; idTarget, predicting possible binding targets of a small chemical molecule via a divide-and-conquer docking approach [18]; and drugCIHPER, using machine learning approach [19]. However, these methods have their own limitations. Chemical similarity search and pharmacophore model cannot obtain high accuracy. Docking approach is restricted by the numbers of targets and computational resources. Only when there is sufficient annotated information as training data and certain amounts of numbers of targets or special chemical space, does machine learning perform well. Such methods are not suitable for large-scale data inference for TCM. Therefore, to obtain more comprehensive each new interaction was considered only and accurate prediction for massive interactions between multi-components and multi-targets, still requires no small effort.

Herein, an approach for large-scale multiple interactions inference as well as TCM network expansion was proposed. We developed a simultaneous interaction prediction approach (SiPA) that combined two essential models, a simple inference model (SIM) that focused on ‘logical relevance’ between compounds, proteins or diseases within interaction network, a compound-target correlation space based interaction prediction model (CTCS-IPM) that calculated the position of compound or protein on the compound-protein correlated space, and more specifically, this space was constructed by canonical correlation analysis (CCA) to predict the vast interactions between multiple compounds, multiple targets and multiple diseases simultaneously for TCM network expansion. In this study, compound *Salvia miltiorrhiza*, also known as Fu-fang Danshen in Chinese, an important prescription with a long history of extensive usage in the treatment of cardiovascular diseases (CVD) [20, 21], was used as model drug to verify the availability of our approach. In practice, the effectiveness of functional modules of the expanded interaction network of compound *Salvia miltiorrhiza*, which was built using a combination of known and predictive compound-target interactions, was thoroughly analyzed.

## Methods

### Data collection and collation

Data related to compound *Salvia miltiorrhiza* including compounds, targets, diseases and their interactions were obtained from public database sources and literatures. Credible compounds were downloaded from Chinese Academy of Sciences Chemical Database (http://chemdb.sgst.cn/scdb/main/find_db.htm), or retrieved from literatures; active targets of the specific compound were obtained from PubChem (https://pubchem.ncbi.nlm.nih.gov/) by searching CAS number of compounds; active targets associated protein–protein interactions were obtained from PharmGKB (https://www.pharmgkb.org/); protein-cardiovascular disease interactions were obtained from OMIM (https://www.omim.org/), and UniProt (https://www.uniprot.org/) was used for retrieving complete protein information. Representations of each data from different sources were unified by mapping to common identifiers, for instance, compounds were represented by general name, alias, CAS, Formula, PubChem CID, and proteins were represented by Entry Gene, Symbol, Gene name, Synonym, HGNC ID, Uniprot ID, and cardiovascular diseases were represented by disease name, OMIM ID, MESH ID. Finally, duplicate or incomplete records were removed according to compound structures and Entry ID respectively. Only those data which have been validated by literatures were considered.

### Simple inference model (SIM)

**Fig. 1:**
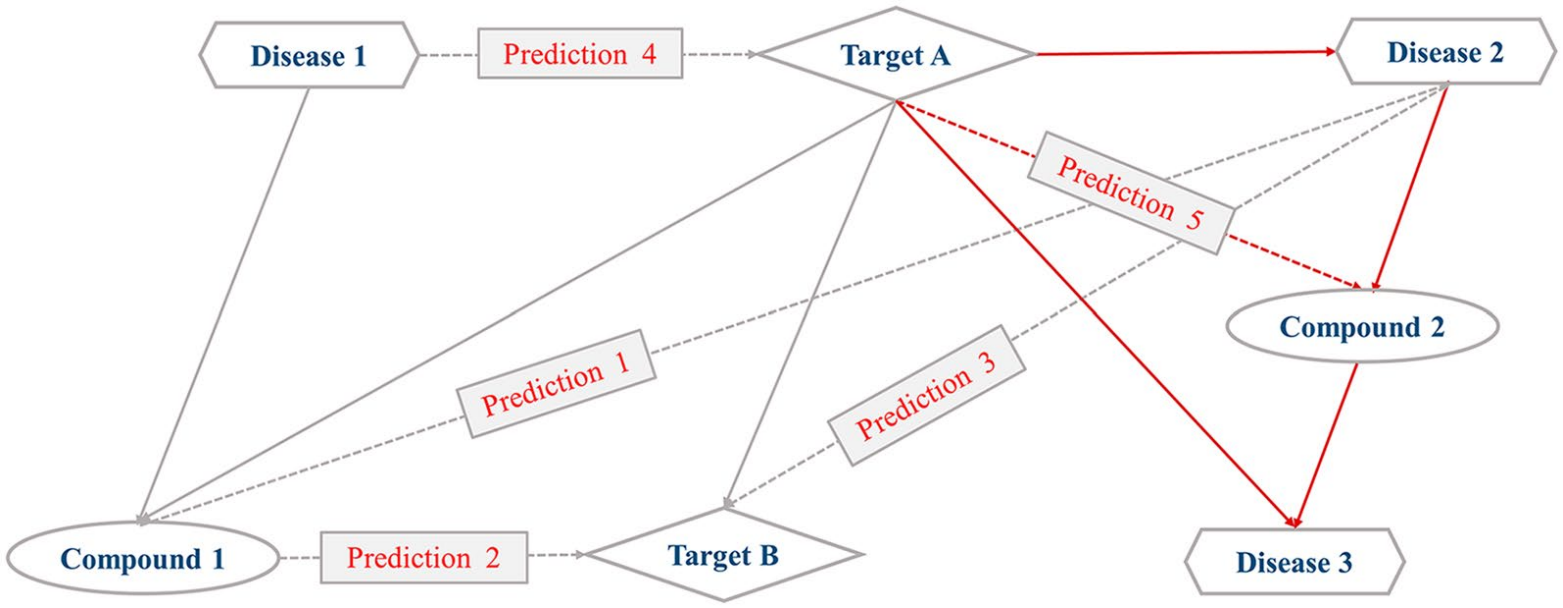
Simple inference model (SIM)

SIM mainly focused on the ‘logical relevance’ between compounds, targets or diseases within interaction network to infer new interactions, on the basis of two threads, targets centered inference and compounds/diseases centered inference, together with following principles (Fig. 1): Principle A, Targets centered inference: 1. If Compound 1 can work on Target A that connects Disease 1, it suggests that Compound 1 can affect Disease 1; 2. If Compound 1 can work on Target A that connects Target B, it suggests that Compound 1 can affect Target B; 3. If Target B can work on Target A that connects Disease 1, it suggests that Target B can affect Disease 1. Furthermore, principle B, Compounds/diseases centered inference: 4. If Compound 1 can interact with Disease 2 and Target A, it provides a possibility that Target A can interact with Disease 2; 5. If Compound 2 can interact with Disease 1 that is related with Target A, it provides a possibility that Compound 2 can interact with Target A. However, compounds/diseases centered inference was still doubtful with more false positive interaction data than that of targets centered inference. Therefore, in order to reduce false positive results caused by compounds/diseases centered inference, each new interaction was considered only when it was inferred more than twice by different known interaction data in a prediction (as shown in Fig. 1, Prediction 5 could be inferred through Disease 2 and Disease 3, respectively). Thus, novel interactions among compounds, targets and diseases could be reliably inferred based on the known interaction data.

### Molecular descriptor selection

Compounds and proteins can be characterized by molecular descriptors, which are the final result of a logical and mathematical process that encoded the chemical information into a useful number or some of the standardized experimental results of the molecular symbol representation [22]. The digitized information provides more insights into the interpretation of the molecular properties and/or is able to take part in a model for the prediction of some interesting properties of other molecules [23].

The compound and protein molecular descriptors were calculated using molecular operating environment (MOE) and ProFeat software, respectively. Subsequently, these molecular descriptors were pre-processed by several criteria to remove redundant data, which not only interfered with the model accuracy, also resulted in the increasing calculation amount and low calculation speed. These removal criteria contain: the molecular descriptors of compound or protein with missing values, the molecular descriptors with reproducibility of values more than 80%, the molecular descriptors with relative standard deviations less than 0.05, and one of the pair of molecular descriptors with the correlation coefficient more than 0.9. Subsequently, feature descriptors were extracted using CCA model based on training data to identify optimal combination of compound and protein descriptors for prediction model.

### Compound-target correlation space based interaction prediction model (CTCS-IPM)

In order to further efficiently explore new interactions between multiple compounds and multiple targets, especially for compounds (or targets) with less interaction information available, CTCS-IPM was established by calculating position of compounds and targets in compound-protein correlation space constructed by CCA. CCA is a multivariate statistical analysis method that uses the correlation between comprehensive variables to reflect overall relevance between the two sets of metrics, providing an effective way to measure the linear relationship between two multidimensional data sets [24, 25]. For two multidimensional variables, it can find the best linear transformation to achieve the maximum correlation between them [26]. Usually, only a few pairs of typical variables can reflect the overall relevance between two variable sets. Here, compound and protein molecular descriptors can be regarded as two variable sets of CCA respectively. Thus, interactions of compounds and proteins can be represented by the correlation between two sets of variables. Typical correlation variables with larger correlation coefficient suggest that the connections between protein and compound, both characterized by these descriptors, are much more closer [27]. Here, CCA was applied using SPSS software (version 20) to calculate the typical correlation coefficient between two variable sets of compound and protein molecular descriptors. Then, these descriptors with larger correlation coefficient were extracted for the characterization of compound and protein space as well as the construction of prediction model.

**Fig. 2:**
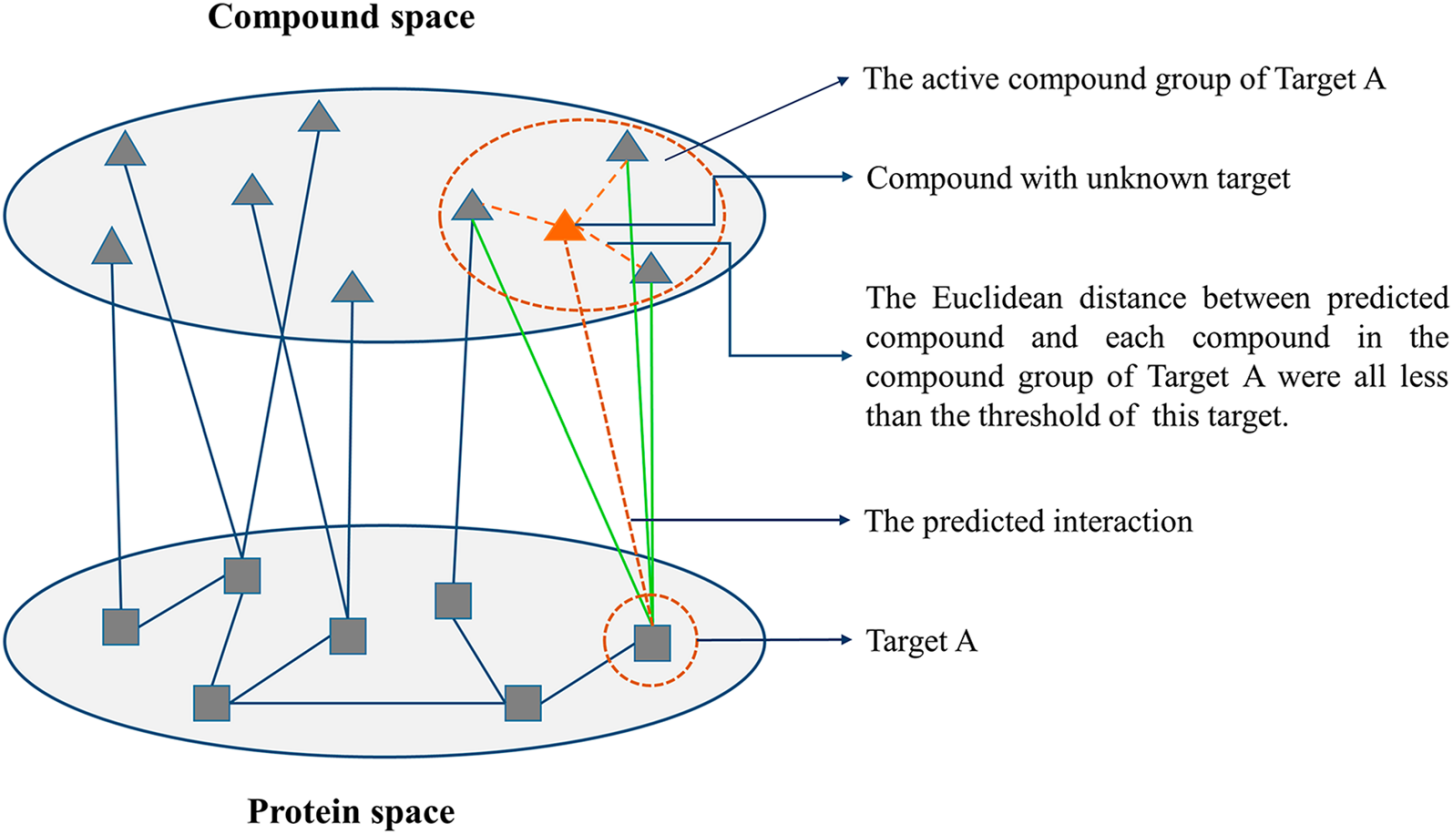
Compound-target correlation space based interaction prediction model (CTCS-IPM)

To predict compound-protein interactions, Euclidean distance, which refers to the real distance between two points in m-dimensional space, or the natural length of the vector, was introduced as a representative measure to define position of compounds or proteins in the compound or protein space respectively. Compounds, acting on the same target in the compound-protein correlation space, would constitute the compound space of the target, vice versa (target space of the compound). For a target (or a compound), Euclidean distances between all compound pairs (or protein pairs) in the compound space of this target (or target space of this compound) were calculated and a threshold of this target (or compound) was defined, which was the upper limit of confidence interval with a 95% confidence level of all distances in the compound space (or target space). Therefore, all targets can have their own threshold in one model. If the Euclidean distance between one compound to be predicted and each compound in the compound space of the target is within the threshold, it is considered that the compound to be predicted could act on the target (Fig. 2). Taken together, the interactions between multiple compounds and multiple proteins could be predicted using CTCS-IPM.

### Interaction prediction and network construction of compound *Salvia miltiorrhiza*

The interactions among compounds, targets (proteins), and cardiovascular diseases were predicted by SiPA. The compound-target interactions predicted using CTCS-IPM were integrated with expansion data obtained from SIM and original known interactions to construct the expanded interaction network. As a contrast, the network only using known interaction data was also constructed. The networks were visualized by Cytoscape software (version 3.7.1) for further analysis.

### Network analysis for original and expanded network of compound *Salvia miltiorrhiza*

Network analysis was considered as an effective way for discovering more potential biological information from the established network. In order to evaluate the effectiveness of our approach, results of network analysis were compared between expanded network and original network on three aspects, including analysis of network overall parameters, analysis of modules from a seed node of specific disease, and analysis of functional modules based on IPCA. To be more specific, IPCA was a clustering algorithm based on the new topological structure, which is robust against the known high rate of false positives and false negatives in data from high-throughput interaction techniques or interaction prediction methods [28]. Finally, the biological activities of partial predicted interactions in the network modules were verified by literatures and molecular docking to prove reliability of our approach. Molecular docking was applied using AutoDock Vina (version 1.1.2) and AutoDock Tools (version 1.5.6).

## Results

### Data collection and collation

After the data preprocessing,192 compounds (Additional file 1: Table S1), including 49 compounds with well-described structure and known targets, 83 compounds with well-described structure but no targets, and the rest 60 compounds without structure and targets, 494 targets (proteins) (Additional file 2: Table S2) and 34 cardiovascular diseases (Additional file 3: Table S3) were collected. On the other hand, 4379 pairs of compound-target interactions (Additional file 4: Table S4) composed of 49 compounds and 398 proteins, 78 pairs of compound-disease interactions (Additional file 5: Table S5) composed of 13 compounds and 15 cardiovascular diseases, 70 pairs of cardiovascular disease-protein interactions (Additional file 6: Table S6) composed of 66 proteins and 23 cardiovascular diseases were obtained. Besides, 47 pairs of protein–protein interactions (Additional file 7: Table S7) were also retrieved. Taken together, these data related to compound *Salvia miltiorrhiza* will be applied in the interaction prediction and network expansion.

### Construction and evaluation of SIM

SIM was constructed based on the ‘logical relevance’ between compounds, targets or diseases within interaction network to infer new interactions. Therefore, new interactions among compounds, targets and diseases could be inferred by identifying common targets. New interactions of disease-target and compound-target could also be inferred by identifying the common neighbor, like compounds or diseases. Since compounds/diseases centered SIM could more likely result in false positive errors as compared to targets centered SIM, its performance was evaluated. Here, known interactions among 5 compounds, 2 targets and 2 cardiovascular diseases with explicit ‘logical relevance’ centered by compounds and diseases were used as test dataset, including 8 pairs of compound-disease interactions involving 5 compounds and 2 cardiovascular diseases, 8 pairs of compound-target interactions involving 5 compounds and 2 targets, and 2 pairs of disease-target interactions involving 2 cardiovascular diseases and 2 targets. Subsequently, in light of these interactions, novel compound-target and disease-target interactions were inferred using principle B. As a result, 8 pairs of compound-target interactions composed of 5 compounds and 2 targets and 4 pairs of disease-target interactions composed of 2 cardiovascular diseases and 2 targets were inferred. These inferred interactions were highly consistent with test dataset, in which 4 pairs of disease-target interactions were inferred more than twice from different interaction routes and two pairs of disease-target interactions were new. These results suggested that the compounds/diseases centered model also had well performance for inferring new potential interactions and effectively reducing false positives (Table 1).

**Table 1:** The predictive performance of compounds/diseases centered SIM

Inferred interactions (Times)	Inferred basis (Known interactions)	Consistent with test set	Inferred interactions (Times)	Inferred basis (Known interactions)	Consistent with test set
C10-T8 (1)	C10-D2-T8	Yes	D12-T13 (3)	D12-C18-T13	Yes
C17-T8 (1)	C17-D2-T8	Yes	D12-T13 (3)	D12-C17-T13	Yes
C18-T8 (1)	C18-D2-T8	Yes	D12-T13 (3)	D12-C29-T13	Yes
C29-T8 (1)	C29-D2-T8	Yes	D2-T8 (5)	D2-C29-T8	Yes
C40-T8 (1)	C40-D2-T8	Yes	D2-T8 (5)	D2-C17-T8	Yes
C17-T13 (1)	C17-D12-T13	Yes	D2-T8 (5)	D2-C10-T8	Yes
C18-T13 (1)	C18-D12-T13	Yes	D2-T8 (5)	D2-C18-T8	Yes
C29-T13 (1)	C29-D12-T13	Yes	D2-T8 (5)	D2-C40-T8	Yes
D12-T8 (3)	D12-C18-T8	New	D2-T13 (3)	D2-C17-T13	New
D12-T8 (3)	D12-C17-T8	New	D2-T13 (3)	D2-C29-T13	New
D12-T8 (3)	D12-C29-T8	New	D2-T13 (3)	D2-C18-T13	New

### SIM based interaction prediction for compound *Salvia miltiorrhiza*

In light of the built SIM, novel interactions among compounds, targets and diseases related to compound *Salvia miltiorrhiza* were predicted using the known interaction data. After the removal of existing and reduplicative data, 24 pairs of compound-cardiovascular disease interactions (Additional file 8: Table S8), 294 pairs of cardiovascular disease-protein interactions (Additional file 9: Table S9) and 191 pairs of compound-target interactions (Additional file 10: Table S10) were obtained.

### Construction and evaluation of CTCS-IPM

Molecular descriptors of 132 compounds with well-described structure in compound *Salvia miltiorrhiza* and 398 targets (corresponding to the 49 compounds) were calculated by MOE and ProFeat, respectively. As a result, 365 original compound molecular descriptors, containing 2D and 3D descriptors in 13 categories, and 1437 original protein molecular descriptors in 9 categories were obtained [29, 30]. Next, with the help of stratified sampling method, 4379 pairs of known compound-target interactions were randomly divided into two groups at the ratio of 4:1 for each target. One group was set as training dataset, 3501 pairs consisting of 49 compounds and 394 targets, and another group was test dataset, 878 pairs consisting of 47 compounds and 380 targets. Then, the preprocessing of molecular descriptors was performed based on training dataset to remove redundant data, showing that 93 compound molecular descriptors and 355 protein molecular descriptors remained for CCA calculation. Here, CCA was applied to calculate the typical correlation coefficient between compound and protein molecular descriptors. Typical correlation variables (the corresponding compound and protein molecular descriptors) with significance less than 0.01 and correlation coefficient greater than 0.8 were chosen as final feature descriptors. Finally, 16 compound molecular descriptors (Table 2) and 42 protein molecular descriptors (Table 3) were extracted to represent the compound space and protein space. Then, the Euclidean distance between each compound or target pair was calculated, and the threshold for a specific compound group of each target was defined. As a consequence, CTCS-IPM was obtained for interactions inference by calculating position of compounds and targets in compound-protein correlation space.

**Table 2:** The selected compound molecular descriptors

Molecular descriptor of compounds	Description
BCUT_SMR_3	Molar Refractivity BCUT (3/3)
b_double	Number of double bonds
b_max1len	Maximum single-bond chain length
dipole	Dipole moment
dipoleX	Dipole moment (X)
dipoleY	Dipole moment (Y)
E_ele	Electrostatic energy
E_vdw	Van der Waals energy
FASA+	Fractional positive accessible surface area
GCUT_SLOGP_2	LogP GCUT (2/3)
GCUT_SMR_0	Molar Refractivity GCUT (0/3)
PEOE_RPC-	Relative negative partial charge
PEOE_VSA + 5	Total positive 5 vdw surface area
PEOE_VSA-0	Total negative 0 vdw surface area
PEOE_VSA-1	Total negative 1 vdw surface area
PEOE_VSA_FPOS	Fractional positive vdw surface area
PEOE_VSA_FPPOS	Fractional polar positive vdw surface area
pmiX	Principal moment of inertia (X)
pmiZ	Principal moment of inertia (Z)
Q_VSA_FPNEG	Fractional polar negative vdw surface area
Q_VSA_FPPOS	Fractional polar positive vdw surface area
rsynth	Synthetic Feasibility

**Table 3:** The selected protein molecular descriptors

Molecular descriptor of proteins	Description	Molecular descriptor of proteins	Description
AL	Dipeptide composition	TR	Dipeptide composition
GA		VD	
GK		VF	
GL		VQ	
GP		VR	
HA		VY	
HL		DL	
IA		EA	
ID		EL	
IL		FL	
IN		M-B (1) by AA index 1	Autocorrelation descriptors
IR		M-B (12) by AA index 1	
PL		M-B (21) by AA index 1	
QL		M-B (23) by AA index 1	
SA		M-B (30) by AA index 1	
SL		M-B (1) by AA index 2	
SP		M-B (10) by AA index 2	
SR		M-B (16) by AA index 2	

Furthermore, this model was evaluated by tenfold cross-validation [31]. As shown in Table 4, the validation result in each round recalled more than 90% of pairs in the test dataset, giving rise to an average recall rate up to 93.56%. This validation result obviously underscored how well our established model to predict the potential interactions. Much more interesting, results based on additional test dataset containing 878 pairs consisting of 47 compounds and 380 targets predicted 1607 pairs of interactions between compounds and targets, in which 818 pairs exactly fitted with test dataset with a recall rate reaching 93.17%, while remaining 789 new interactions lacked reference. It’s proved that the CTCS-IPM had a very good predictive performance.

**Table 4:** Validated performance of the CTCS-IPM

Number	The number of pairs in training dataset	The number of pairs in test dataset	The number of predicted pairs consistent with test dataset	Recall rate (%)
1	3145	358	330	92.18
2	3145	358	329	91.90
3	3152	351	335	95.44
4	3159	344	320	93.02
5	3157	346	327	94.51
6	3156	347	332	95.68
7	3147	356	328	92.13
8	3153	350	331	94.57
9	3155	348	324	93.10
10	3158	345	321	93.04
average	3153	350	328	93.56

### CTCS-IPM based interaction prediction for compound *Salvia miltiorrhiza*

In this study, interactions between 132 compounds with identified structure and 398 proteins were simultaneously predicted by CTCS-IPM. As a result, 519 pairs of new compound-target interactions were predicted (Additional file 11: Table S11). Among them, 238 pairs of interactions consisting of 63 proteins and 25 compounds without any previous target information were also successfully predicted. In addition, most compounds could interact with more than one target, for example, Alexandrin could act on 43 various targets (Table 5). The new interactions predicted by SIM and CTCS-IPM were then integrated. After the removal of reduplicative interactions, 710 pairs of new compound-target interactions, 24 pairs of new compound-cardiovascular disease interactions and 294 pairs of new cardiovascular disease-protein interactions were obtained for expanding the network of compound *Salvia miltiorrhiza*.

**Table 5:** Numbers of predicted targets of compounds without any previous target information

Phytometabolites	Compounds	Number of predicted targets	Phytometabolites	Compounds	Number of predicted targets
Flavonoids	Alexandrin	43	Glycosides	d-Glucose	
	Miltipolone	1		Eleutheroside A	
	Salvilenone	2		Ginsenoside-Rh1	
	Salviolone	9		Gypenoside VIII	
	Tanshinaldehyde	2		Gypenoside III	
	Tanshinone IIB	2		Gypenoside XVII	
	Tigogenin	43	Phenyl methane	Dicapryl Phthalate	14
Volatile oil	Cuparene	3	Hydrocarbon	Docosane	2
Terpenoids	Cyperene	1		Ethyl Octadecadienoate	2
	α-Gurjunene	5		Non-3-En-2-One	1
	α-Muurolene	4	Nitrogenous	Dencichine	1
	β-Cubebene	18	Nonsteroidal	Stigmasterol	43
	γ-Cadinene	3			

### Network construction of compound *Salvia miltiorrhiza*

The original compounds-targets-cardiovascular diseases interaction network (original network) was constructed using initial collected data; meanwhile the expanded network was built in a similar way on the basis of the integrated data of original collated and predicted interactions of compound *Salvia miltiorrhiza*. To more explicitly analyze the context of the networks, they were visualized by Cytoscape software. The original network consisted of 577 nodes and 4574 edges, containing 49 compounds with known targets, 494 proteins and 34 cardiovascular diseases (Additional file 12: Figure S1), while expanded network increased compound amount up to 74, consisting of 602 nodes and 5602 edges (Additional file 12: Figure S2).

### Network analysis for original and expanded network

To assess the influence of predicted interactions on TCM network in the content, original and expanded networks were analyzed on three aspects, including the parameters of overall network, specific diseases centered modules as well as analysis of functional modules, respectively. Then, biological activities of partial predicted interactions in the network modules were verified by literatures and molecular docking to prove reliability of SiPA.

#### Comparison of parameters between original and expanded network

The parameters mainly reflected the typical topology properties of networks; therefore, the difference of the parameter values between the original and expanded network was investigated (Table 6). In the original network, the average number of adjacent nodes was 15.854, revealing the complex network relationship among compounds, proteins and cardiovascular diseases. The length of the characteristic path in the network was 2.963, which indicated that any two nodes in the network could be connected by no more than three nodes, embodying the “small world” of biological network. The network diameter was 8, indicating that two most distant nodes in the network could be connected through eight nodes. By comparison, the density of expanded network increased from 0.026 to 0.289, and the network diameter and characteristic path length were shortened, which suggested that nodes in expanded interaction network connected more closely. Heterogeneity parameter of expanded network was reduced by 1.622 than that of original network, indicating that the expended network was easier to achieve homogeneity. In addition, characteristic path length in expanded network was narrowed from 2.963 to 1.774. These results showed that interactive relations among compounds, targets and cardiovascular diseases were effectively complemented and the expanded compounds-targets-cardiovascular diseases interaction network had a higher integrity as expected.

**Table 6:** Parameters of original network and expanded network

Parameters	Values of original network	Values of expanded network
Number of nodes	577	602
Number of edges	4574	5602
Connected components	9	1
Network diameter	8	3
Network radius	1	2
Network density	0.026	0.289
Network heterogeneity	2.502	0.880
Network centralization	0.539	0.731
Characteristic path length	2.963	1.774
Avg. number of neighbors	15.854	5.5
Isolated nodes	0	0

#### Analysis of specific disease centered modules

To further investigate whether the expanded interaction data can significantly improve network integrity and provide effective information for the understanding of the mechanism of compound *Salvia miltiorrhiza* on specific cardiovascular diseases, disease centered network modules were extracted from original and expanded interaction network respectively and subsequently analyzed.

Firstly, Diabetes Mellitus Type 1 (D23) was used as a seed node to determine a suitable path length that could properly distinguish representative information on the original and expanded network to limit the size of extracted modules. When the path length was set as 1, the modules showed the closest targets or compounds to the seed node (D23), lacking of comprehensive representation of interactions among compounds, targets and diseases. Although target interaction information was complemented in expanded module (Additional file 12: Figure S3a) compared with original module (Additional file 12: Figure S3b), neither of the modules extracted from original and expanded network contained compounds. Thus, it was expected to increase the path length to get more interaction information. The average path of the original or expanded network was between 2 and 3. When the path length was equal to or greater than 3, more comprehensive interactions related to the seed node and more redundancy information would be contained in the mined module (Additional file 12: Figure S4). Accordingly, in order to extract the disease centered module that could more completely describe regulation information among compounds, proteins and diseases and effectively reduce information redundancy, the path length, in this study, was set as 2 for module mining from original and expanded network.

As a result, 34 cardiovascular diseases centered modules were extracted with path length of 2 from original and expanded network respectively (Table 7). Interactions were increased in most expanded modules. For example, there were 24 pairs of new direct compound-cardiovascular disease interactions involving 9 compounds and 8 diseases, 8 pairs of which were verified by literatures (Table 8). Furthermore, aiming to systematically investigate the relationship among compounds, targets and diseases in disease centered modules, these modules focused on three representative cardiovascular diseases, Diabetes mellitus Type 1, QTL regulation of blood pressure, and Long QT syndrome 4 were further analyzed.

**Table 7:** Comparison of original and expanded modules focusing on specific disease

	Original modules			Expanded modules		
	Compounds	Diseases	Targets	Compounds	Diseases	Targets
D1	8	14	316	27	22	348
D2	7	14	318	50	22	349
D3	5	14	315	27	22	347
D4	3	13	313	38	23	346
D5	8	14	317	27	22	349
D6	8	14	316	27	22	348
D7	9	14	317	28	23	349
D8	2	12	313	27	22	345
D9	5	12	315	27	22	347
D10	5	14	316	27	22	348
D11	6	14	316	28	23	348
D12	5	12	15	8	20	356
D13	3	11	6	8	16	17
D14	5	14	315	27	22	347
D15	1	2	12	1	2	13
D16	1	1	1	1	3	1
D17	5	1	19	5	21	359
D18	2	1	9	2	20	348
D19	5	2	23	5	20	365
D20	5	2	2	5	20	348
D21	2	2	1	2	19	342
D22	2	2	1	2	19	342
D23	0	1	5	2	14	17
D24	0	1	1	0	1	1
D25	0	1	4	2	14	4
D26	0	2	1	0	2	1
D27	0	1	1	0	1	1
D28	0	1	2	0	1	2
D29	0	1	1	0	1	1
D30	0	1	1	0	1	1
D31	0	2	1	0	2	1
D32	0	2	1	0	2	1
D33	0	1	5	0	1	5
D34	0	0	1	0	0	1

**Table 8:** Literature verification of predicted direct compound-cardiovascular disease interactions

Predicted interactions	Validated literatures
Borneol (C8)—Hyperlipidemia (D16)	Borneol has ameliorative effect of hyperlipidemia in diabetic Wistar rats [32]
Cryptotanshinone (C10)—Diabetes mellitus type 2 (D20)	Cryptotanshinone has effect of antidiabetes via activation of AMP-activated protein kinase [33]
Ginsenoside-Rg1 (C18)—Diabetes mellitus type 2 (D20)	Ginsenoside-Rg1 can alleviate the insulin resistance through increasing the uptake of glucose and decreasing the output of glucose [34, 35]
Tanshinone IIA (C40)—Diabetes mellitus type 2 (D20)	Tanshinone IIA may alleviate type 2 DM symptoms in experimental rats [36]
Cryptotanshinone (C10)—Obesity (D12)	Cryptotanshinone promotes commitment to the brown adipocyte lineage and mitochondrial biogenesis in C3H10T1/2 mesenchymal stem cells to alleviate obesity [37]
Tanshinone IIA (C40)—Obesity (D12)	Tanshinone IIA may treat obesity through PPARγ [38]
Tanshinone IIA (C40)—Angina pectoris (D18)	Sodium tanshinone IIA silate can act as an add-on therapy in patients with unstable angina pectoris [39]
Ginsenoside-Rg1 (C18)—Acute Myocardial infarction (D1)	Ginsenoside-Rg1 could enhance angiogenesis and ameliorates ventricular remodeling in a rat model of Acute Myocardial infarction [40]

**Fig. 3:**
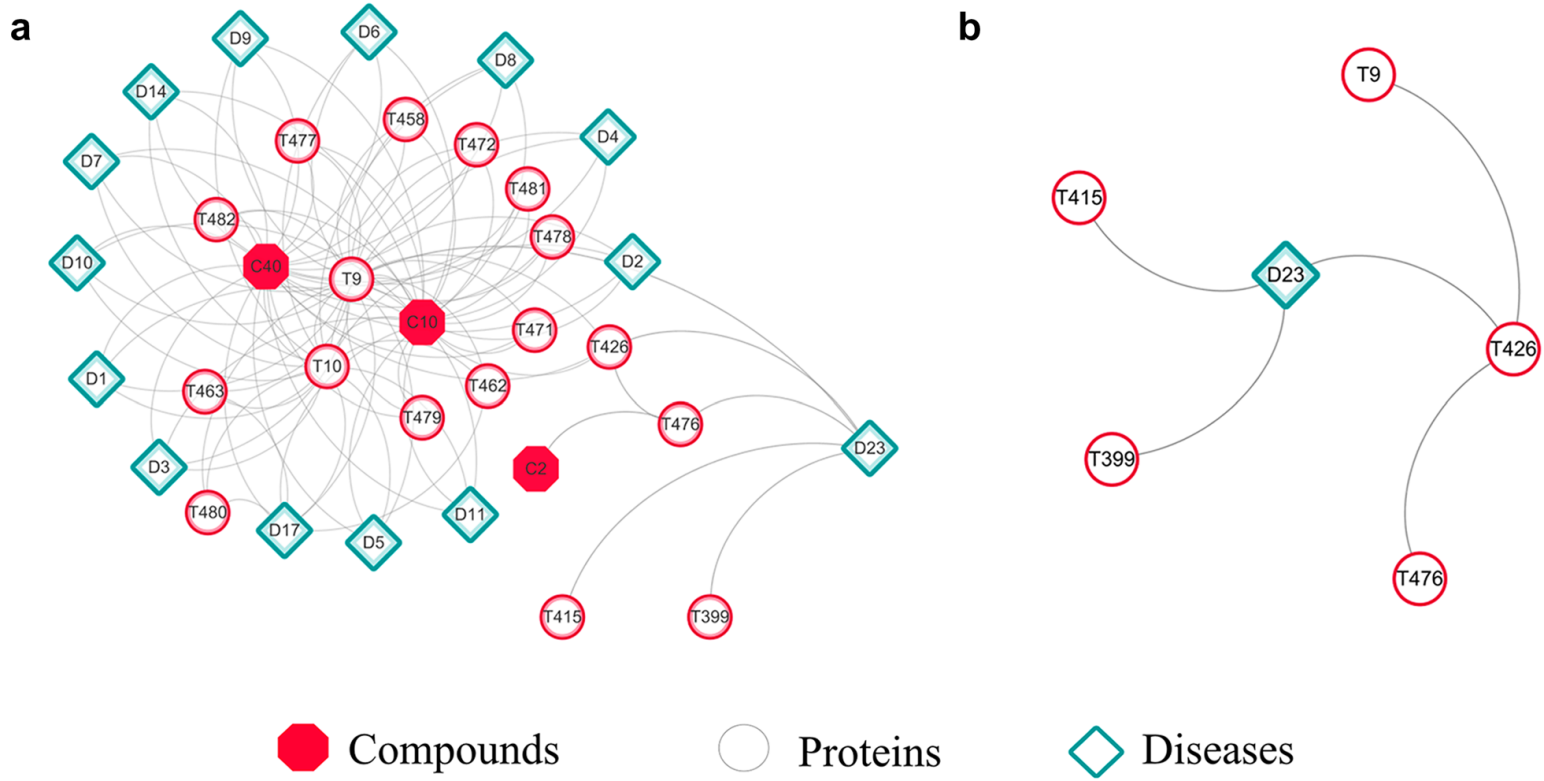
D23 centered modules generated from expanded (**a**) and original (**b**) network

*Diabetes mellitus Type 1 centered modules.* The modules focused on Diabetes Mellitus Type 1 (D23) was excavated with path length of 2 from original and expanded compound *Salvia miltiorrhiza* interaction network respectively. There were three compounds, 2α-Hydroxy Ursolic Acid (C2), Cryptotanshinone (C10) and Tanshinone IIA (C40), associated with D23 through Insulin receptor substrate 1 (T10) and Insulin-degrading enzyme (T476) indirectly in the expanded module (Fig. 3a), while no compound was included in the original module (Fig. 3b). In addition, C10 and C40 could also associate with 13 other cardiovascular diseases, such as Hyperinsulinemic Hypoglycemia (D8), Coronary heart disease (D17), through common targets of T9 and T10. Compared with the original module, proteins increased from 5 to 17 in the expanded module. 2′-5′-oligoadenylate synthase 1 (T399), FOXP3 protein (T415), Insulin receptor substrate 2 (T426) could connect to D23 directly and Insulin-degrading enzyme (T476), Insulin receptor (T9) could affect D23 indirectly in the original module, while the expanded module showed that all above targets connected to D23 directly.

In order to further validate the effects of above three compounds (C2, C10 and C40) on Diabetes Mellitus Type 1 (D23), literatures verification was carried out. It was reported that 2α-Hydroxy Ursolic Acid (C2) could reduce blood glucose in hereditary diabetic mice [41]. Furthermore, results of molecular docking showed that C2 could bind to Insulin receptor substrate 1 (T10) (Additional file 12: Figure S5).

**Fig. 4:**
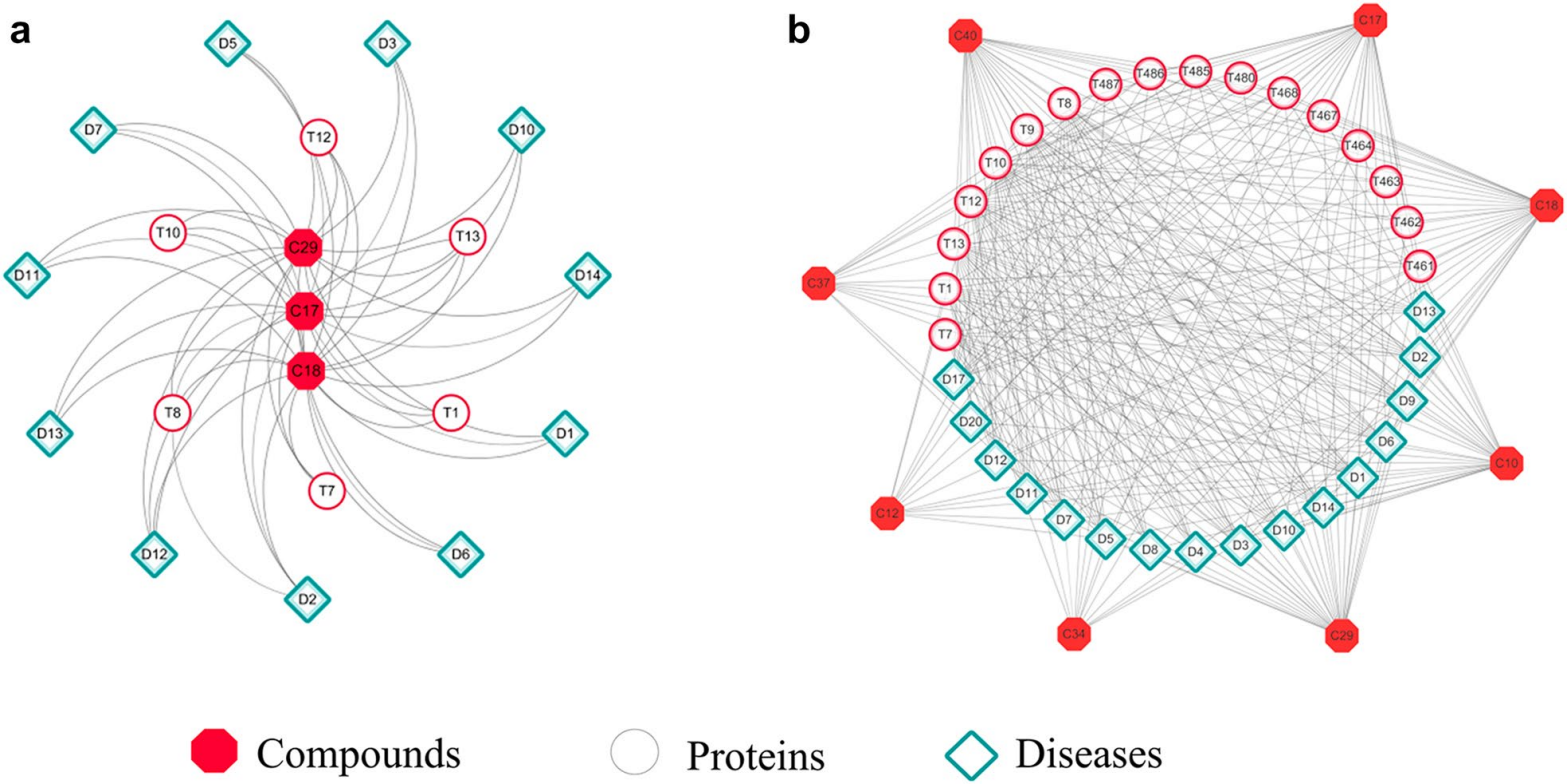
D13 centered modules generated from original (**a**) and expanded (**b**) network

*QTL regulation of blood pressure centered modules.* The modules focused on QTL Regulation of Blood Pressure (D13) were extracted with path length of 2 from original and expanded network respectively. In the original module (Fig. 4a), Gensenoside-Rb1 (C17), Ginsenoside-Rg1 (C18), Notoginsenoside-R1 (C29) could interact with D13 directly. By comparison, in addition to direct interactions between C17, C18, C29 and D13, Cryptopanshinone (C10), Danshengsu (C12), Protocatechuic Aldehyde (C34), Salvianolic Acid B (C37), Tanshinone IIA (C40) also associated to D13 through Angiotensin I converting enzyme (T1), E-selectin (T7), Insulin receptor substrate 1 (T10), Nitric oxide synthase, endothelial (T12) and Estrogen receptor (T486) in the expanded module(Fig. 4b). Besides, all compounds also connected to other 15 cardiovascular diseases directly, such as Hyperinsulinemic Hypoglycemia (D8), Coronary heart disease (D17).

Subsequently, literatures verification showed that salvianolic acid B (C37) could reduce the expression of PLAT protein, enhance cell fibrinolysis and reduce cell adhesion to inhibit blood thrombosis and atherosclerotic plaque formation, which helped maintain the normal arterial blood pressure [42]. Results of molecular docking showed that Danshengsu (C12) could bind to Estrogen receptor (T486) (Additional file 12: Figure S6).

**Fig. 5:**
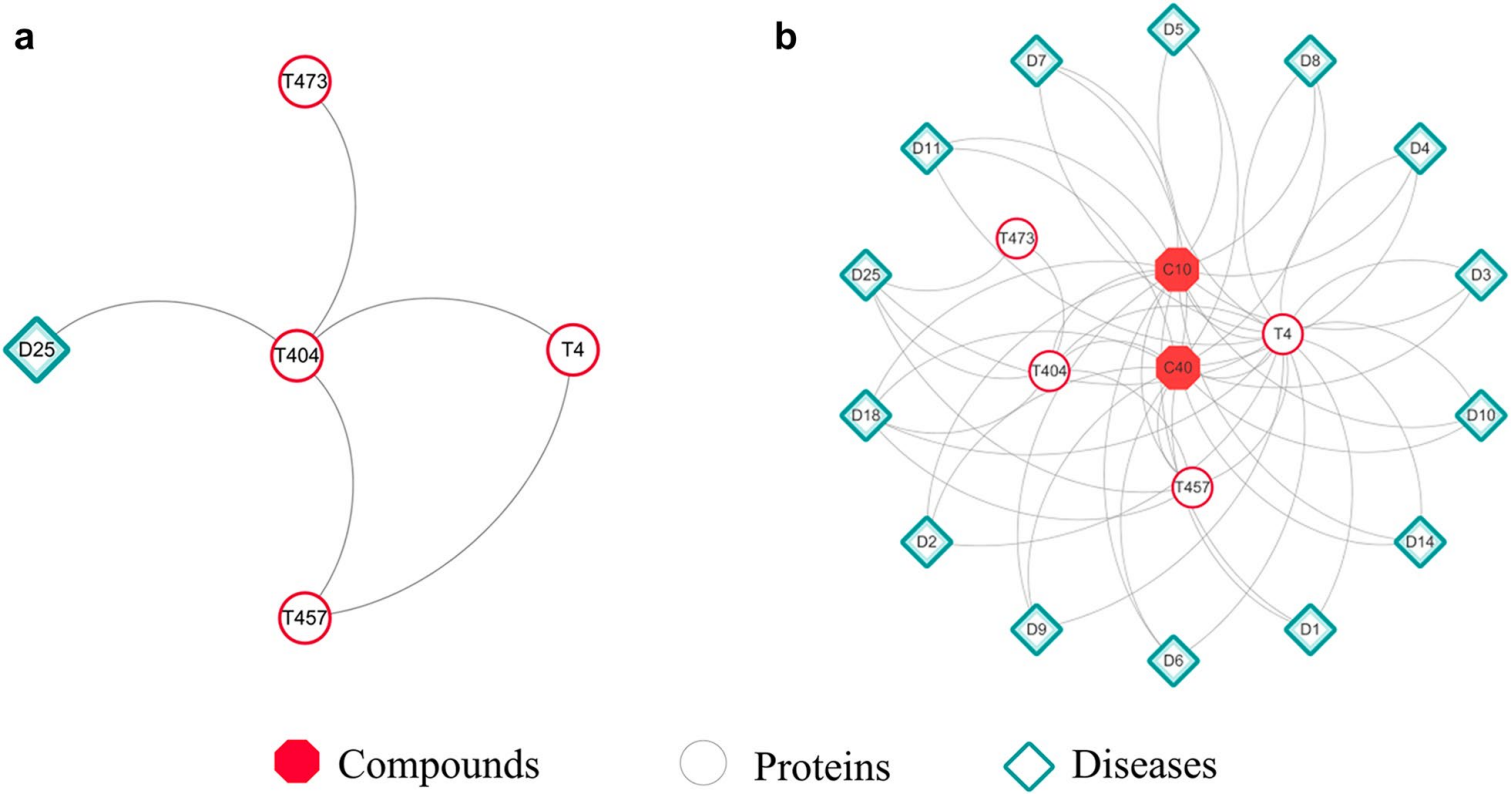
D25 centered modules generated from original (**a**) and expanded (**b**) network

*Long QT Syndrome 4 centered modules.* The Long QT Syndrome 4 (D25) was also used as the seed node to excavate the modules at the path length of 2 from original and expanded network. Only ATP-sensitive inward rectifier potassium channel 11 (T4), Ankyrin-2 (T404), Sodium/calcium exchanger 1 (T473) and ATP-binding cassette sub-family C member 8 (T457) were contained in original module without any more information of compounds (Fig. 5a), while more complete interactions among D25, targets and compounds were included in the expanded module (Fig. 5b). The expanded module showed that Cryptotanshinone (C10) and Tanshinone IIA (C40) might affect D25 through T4, T404 and T457 and connect other 13 cardiovascular diseases directly, such as Angina pectoris (D18).

Similarly, literatures verification and molecular docking were carried out. Although new interactions related to D25 have not been verified, previous study has showed that sodium Tanshinone IIA silate (C40) might have protective effects on Angina pectoris (D18) as an add-on therapy in patients, which is in accordance with the predicted result in this study [39]. Results of molecular docking showed that C40 could bind to ATP-sensitive inward rectifier potassium channel 11 (T4) (Additional file 12: Figure S7).

#### Functional module analysis of compound Salvia miltiorrhiza interaction network

**Fig. 6:**
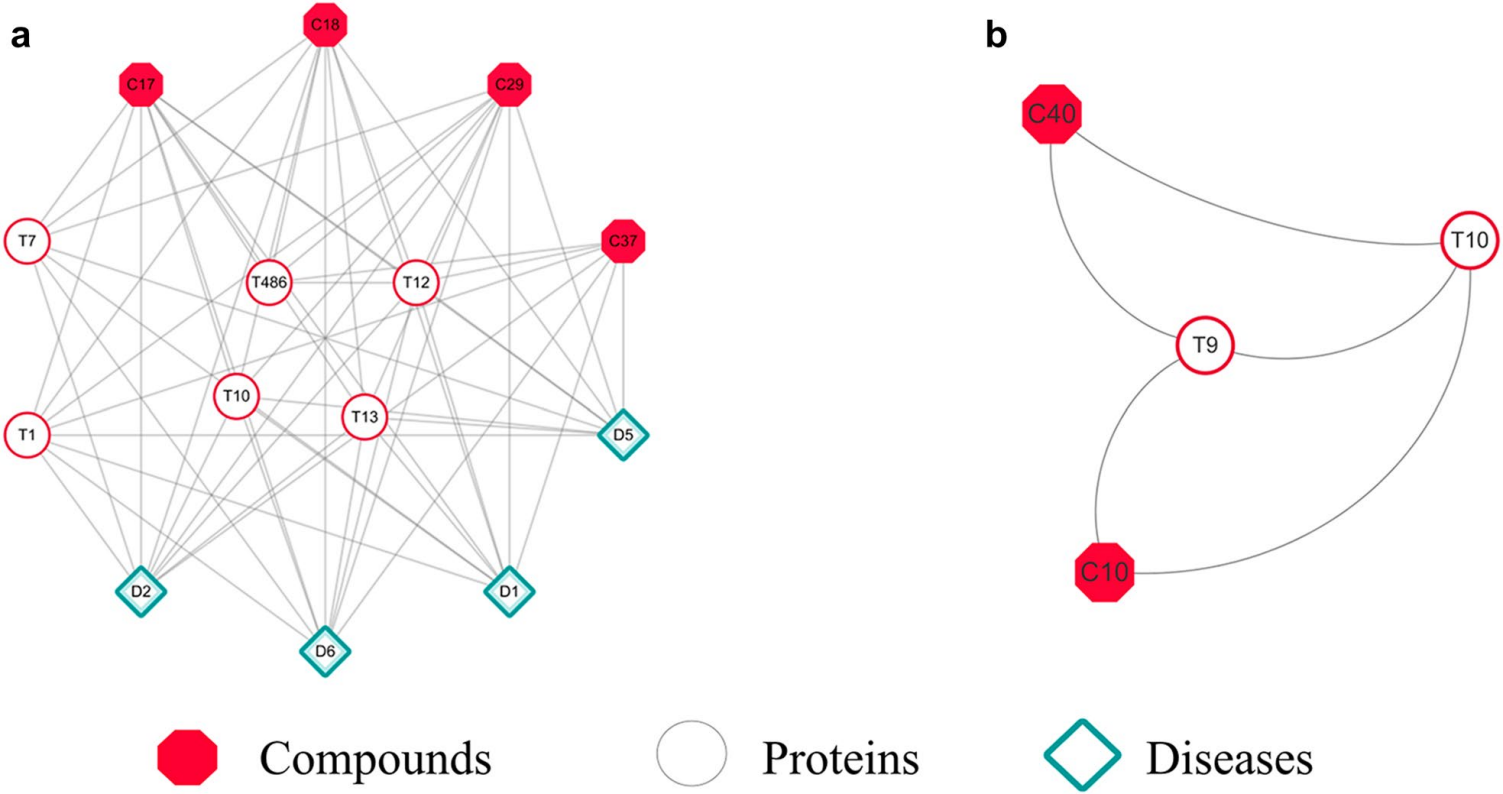
The important modules identified from expanded (**a**) and original (**b**) network

To further evaluate whether expanded network can provide useful functional modules to help discover novel knowledge, the Identifying Protein Complex Algorithm (IPCA) was used to analyze the network, more specifically, to compare differences between original and expanded functional modules. Because of the fact that modules with small node numbers are generally of less importance in the network analysis, the minimum number of nodes in the setup module was set as 14 in this study. No module with nodes equal to or more than 14 was identified in original network, and the number of nodes in maximum functional module was only 4. Instead, 22 modules were dug out in expanded network, and one module with nodes equal to 14, which involved Acute Myocardial Infarction (D1), Atherosclerosis (D2), Coronary Artery Disease (D5) and Diabetic Microangiopathy (D6) (Fig. 6a). Further analysis of this functional module showed that compounds Cryptotanshinone (C10), Gensenoside-R b1 (C17), Ginsenoside-Rg1 (C18), Notoginsenoside-R1 (C29), Salvianolic Acid B (C37) and proteins Angiotensin I converting enzyme (T1), E-selectin (T7), Insulin receptor substrate 1 (T10), Nitric oxide synthase, endothelial (T12), Peroxisome proliferator-activated receptor (T13), Estrogen receptor (T486) could connect to D1, D2, D5, and D6 directly. However, no significant cardiovascular diseases were found in the maximum module from original network (Fig. 6b).

Subsequently, literatures verification showed that inhibiting Angiotensin I converting enzyme (T1) could reduce mortality and the occurrence of severe left-ventricular dysfunction of Acute Myocardial Infarction (D1) patients [43] and Ginsenoside-Rg1 (C18) could enhance angiogenesis and ameliorates ventricular remodeling in a rat model of Acute Myocardial Infarction (D1) [41]. Results of molecular docking further validated that Cryptotanshinone (C10) could bind to Estrogen receptor (T486) (Additional file 12: Figure S8).

In sum, the evaluation using both test dataset suggested that the combination approach showed pretty good performance on accurate interaction prediction. Furthermore, results of network analysis indicated that in light of the integrated interactions, the network expansion could dramatically improve the completeness of the network of compound *Salvia miltiorrhiza*, while the original network only described monotonous interactions without systematic relations among compounds, targets and diseases. Although it was difficult to verify all results of prediction and network analysis, our results of literature validation and molecular docking concluded that this approach had good reliability, and could provide more useful information for exploring the mechanism of compound *Salvia miltiorrhiza* on cardiovascular diseases. Therefore, our attempt to develop a large-scale interaction prediction approach for TCM network expansion is a bit more successful for more comprehensively understanding the mechanism of TCM and for better application of TCM in disease prevention and treatment.

## Discussion

SiPA offered three prominent advantages. Firstly, note that the majority of state-of-art interaction inference methods would lack prediction power without annotated information as negative samples. Negative information of compound-target interaction is extremely limited, so acquisition of reliable negative samples is challenged. However, SiPA was established without negative samples to avoid this limitation, and demonstrated large capability of simultaneously predicting reliable interactions between multiple compounds, diverse targets and various diseases, making it a powerful enough approach to reduce prediction error associated with unreliable negative samples. Secondly, CTCS-IPM could be applied to various challenging scenarios: predicting from small samples with high accuracy, which always failed to construct prediction model by other state-of-the-art methods, and more important, inferring the large-scale interactions of TCM ingredients which always have less, even no known compound-target information available. Although a large number of abundant biomedical data have been accumulated, compound-target interaction information is still inadequate, and applied, in most of the cases, for the investigation of low molecular weight chemicals. Thirdly, SiPA was not restricted by target 3D structures as compared to molecular docking, which could also be applied in large-scale interactions inference. Herein, SiPA provided a practical and efficient way for large-scale inference of multiple interactions of TCM ingredients.

According to previous reports, most current existing interaction prediction models could only infer single type of interactions, like protein–ligand or disease-target interactions. Other models constructed by molecular descriptors, for example, chemogenomics based methods [44, 45], showed the capability to infer interactions of multiple compounds and multiple proteins simultaneously and a higher prediction accuracy compared with CTCS-IPM. More specifically, such better prediction performance of these models should heavily rely on similarity measures of drugs and proteins; therefore, these models would fail in the prediction of TCM because of the diverse targets of TCM ingredients. The Similarity Ensemble Approach (SEA) was also suitable for inferring multiple compound-target interactions through evaluating receptors similarity [14]. However, SEA suffered from the problem of the activity cliff, which is defined as pairs of structurally similar molecules with large differences in potency [46], and was failed to infer new interactions for compounds without well-described structures. CTCS-IPM defined compound-target correlated space based on CCA and a statistical threshold to consider diversity of compounds, which not only estimated the activity cliff, also absorbed features of compounds with large differences in potency for more appropriate inference. When using the SiPA, with the help of network analysis algorithm, more unreliable information was filtered out within the inferred unexpected interactions. Moreover, results of literature and molecular docking have validated the reliability of predicted interactions. Collectively, our SiPA could reach the sufficiently high performance on the prediction of the complicated interaction network of TCM.

TCM is becoming a rich resource for candidate drugs. So appropriate approaches to thoroughly comprehend TCM interactions is particularly important, as it facilitates the identification of potential novel drug leads and advances the quick hit-to-lead development from TCM. SiPA provided a possibility for more effective study of TCM using network pharmacology, and could be applied to effectively identify compound (or targets) candidates in drug discovery.

## Conclusions

In this study, we first proposed a combination approach (SiPA) of SIM centered on the definition of ‘relevance’ between compounds, targets or diseases within interaction network and CTCS-IPM based on the position of compounds and targets in compound-protein correlated space to infer large-scale multiple interactions for understanding the synergistic mechanism of TCM. This approach was successfully applied to predict 710 pairs of new compound-target interactions, 24 pairs of new compound-cardiovascular disease interactions and 294 pairs of new cardiovascular disease-protein interactions for the TCM compound *Salvia miltiorrhiza*. Compound-target interactions were also obtained for 26 compounds without known target information available.

It’s noteworthy that we also applied the expanded network to explore the mechanism of TCM for the first time. Since the completeness of the interaction network was substantially improved, the expanded network modules had a well description on relations of compounds, targets and diseases thoroughly and systematically, offering new insights into underlying mechanism of TCM. As a result, our approach could more comprehensively and explicitly expound the active ingredients of compound *Salvia miltiorrhiza* and their network synergistic mechanism on specific cardiovascular diseases.

In addition, the CTCS-IPM was currently restricted to predict interactions between compounds and targets. To unleash the full potential of the CTCS-IPM, it can be further extended to predict interactions between proteins and diseases or between compounds and diseases by defining an appropriate disease space in future research.

## **Supplementary information**

**Additional file 1: Table S1**. Compounds in compound *Salvia miltiorrhiza*.

**Additional file 2: Table S2.** Targets (Proteins) related to compound *Salvia miltiorrhiza*.

**Additional file 3: Table S3.** Cardiovascular diseases related to compound *Salvia miltiorrhiza*.

**Additional file 4: Table S4.** Compound-target interactions related to Compound *Salvia miltiorrhiza*.

**Additional file 5: Table S5.** Compound-disease interactions related to Compound *Salvia miltiorrhiza*.

**Additional file 6: Table S6.** Cardiovascular disease-protein interactions related to compound *Salvia miltiorrhiza*.

**Additional file 7: Table S7.** Protein-protein interactions related to compound *Salvia miltiorrhiza*.

**Additional file 8: Table S8**. Compound-cardiovascular disease interactions predicted based on simple inference model.

**Additional file 9: Table S9.** Cardiovascular disease-protein interactions predicted based on simple inference model.

**Additional file 10: Table S10.** Compound-target interactions predicted based on simple inference model.

**Additional file 11: Table S11.** Compound-target interactions predicted based on compound-target correlation space based interaction prediction model.

**Additional file 12: Figure S1.** The original compounds-targets-cardiovascular diseases interaction network of compound *Salvia miltiorrhiza*. **Figure S2.** The expanded compounds-targets-cardiovascular diseases interaction network of compound *Salvia miltiorrhiza.* **Figure S3.** D23 centered modules with path length of 1 mining from expanded (**a**) and original (**b**) network. **Figure S4.** D23 centered module with path length of 3 mining from expended network. **Figure S5.** Molecular docking results of 2α-Hydroxy Ursolic Acid (C2)-Insulin receptor substrate 1 (T10). PDB ID: 5U1M; Binding affinity: −6.5kcal/mol; Residues of H-Bound: ASN178. **Figure S6.** Molecular docking results of Danshengsu (C12)-Estrogen receptor (T486). PDB ID: 3OS8; Binding affinity: −6.2kcal/mol; Residues of H-Bound: ARG394. **Figure S7.** Molecular docking results of Tanshinone IIA (C40)-ATP-sensitive inward rectifier potassium channel 11 (T4). PDB ID: 6C3O; Binding affinity:−7.7kcal/mol; Residues of H-Bound: LYS185. **Figure S8.** Molecular docking results of Cryptotanshinone (C10)-Estrogen receptor (T486). PDB ID: 3OS8; Binding affinity: -7.9kcal/mol; Residues of H-Bound: LEU346.

## Data Availability

All data generated or analysed during this study are included in this published article and its supplementary information files.

## Abbreviations

TCM: Traditional Chinese Medicine
SEA: Similarity Ensemble Approach
PPI: Protein-protein interaction
CVD: Cardiovascular diseases
CCA: Canonical correlation analysis
MOE: Molecular operating environment
SIM: Simple inference model
CTCS-IPM: Compound-target correlation space based interaction prediction model
IPCA: Identifying Protein Complex Algorithm
