# Supplementary material for: Development of simultaneous interaction prediction approach (SiPA) for the expansion of interaction network of traditional Chinese medicine
Source: Chin Med. 2020 Aug 26;15:90. doi: 10.1186/s13020-020-00369-z (PMC7448979; doi:10.1186/s13020-020-00369-z)
Supplement: Supplementary file 12 — Additional file 12: Figure S1. The original compounds-targets-cardiovascular diseases interaction network of compound Salvia miltiorrhiza. Figure S2. The expanded compounds-targets-cardiovascular diseases interaction network of compound Salvia miltiorrhiza. Figure S3. D23 centered modules with path length of 1 mining from expanded (a) and original (b) network. Figure S4. D23 centered module with path length of 3 mining from expended network. Figure S5. Molecular docking results of 2α-Hydroxy Ursolic Acid (C2)-Insulin receptor substrate 1 (T10). PDB ID: 5U1M; Binding affinity: −6.5kcal/mol; Residues of H-Bound: ASN178. Figure S6. Molecular docking results of Danshengsu (C12)-Estrogen receptor (T486). PDB ID: 3OS8; Binding affinity: −6.2kcal/mol; Residues of H-Bound: ARG394. Figure S7. Molecular docking results of Tanshinone IIA (C40)-ATP-sensitive inward rectifier potassium channel 11 (T4). PDB ID: 6C3O; Binding affinity:−7.7kcal/mol; Residues of H-Bound: LYS185. Figure S8. Molecular docking results of Cryptotanshinone (C10)-Estrogen receptor (T486). PDB ID: 3OS8; Binding affinity: -7.9kcal/mol; Residues of H-Bound: LEU346. [file 13020_2020_369_MOESM12_ESM.doc]

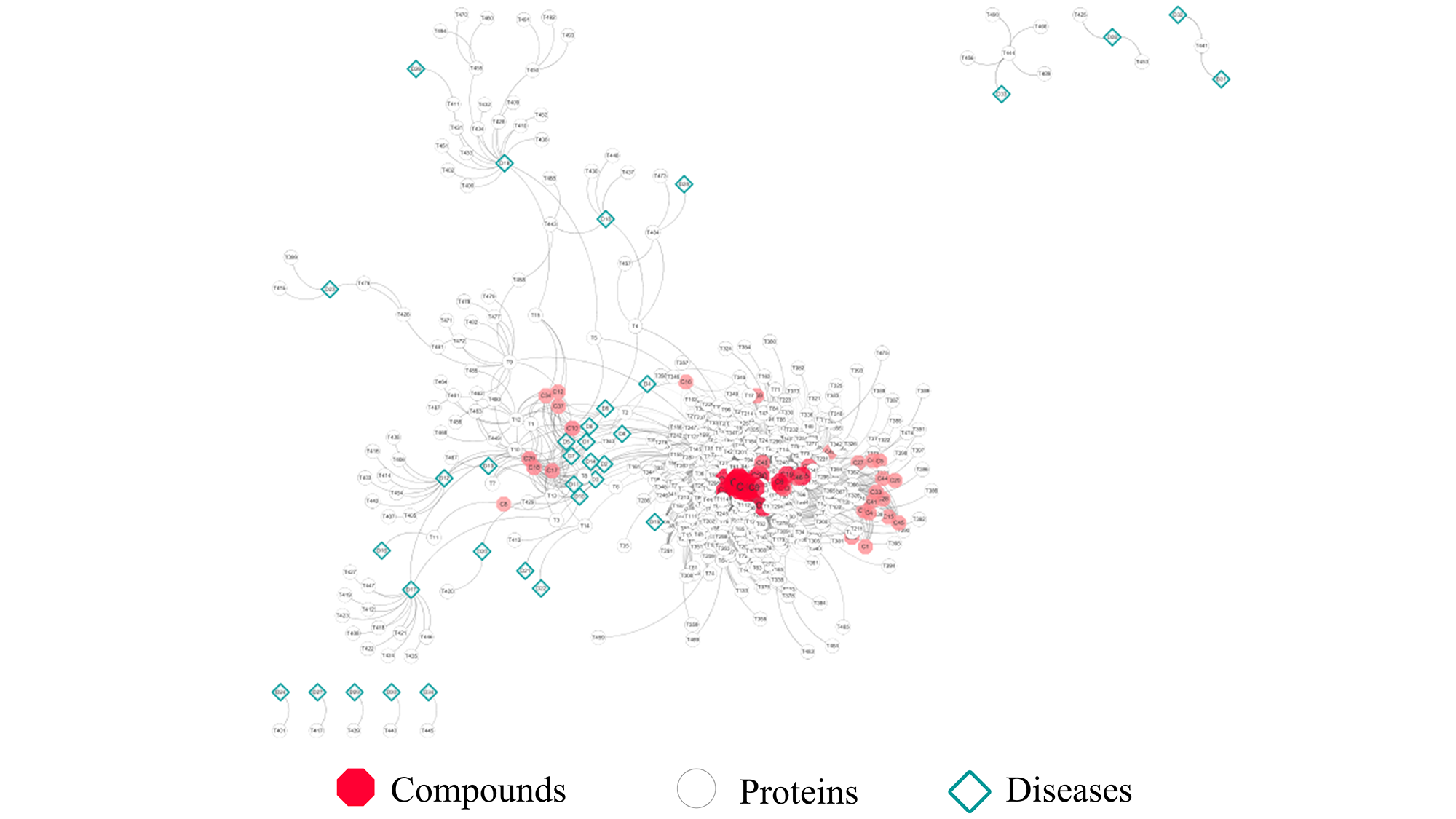


Fig. S1. The original compounds-targets-cardiovascular diseases interaction network of compound *Salvia miltiorrhiza*


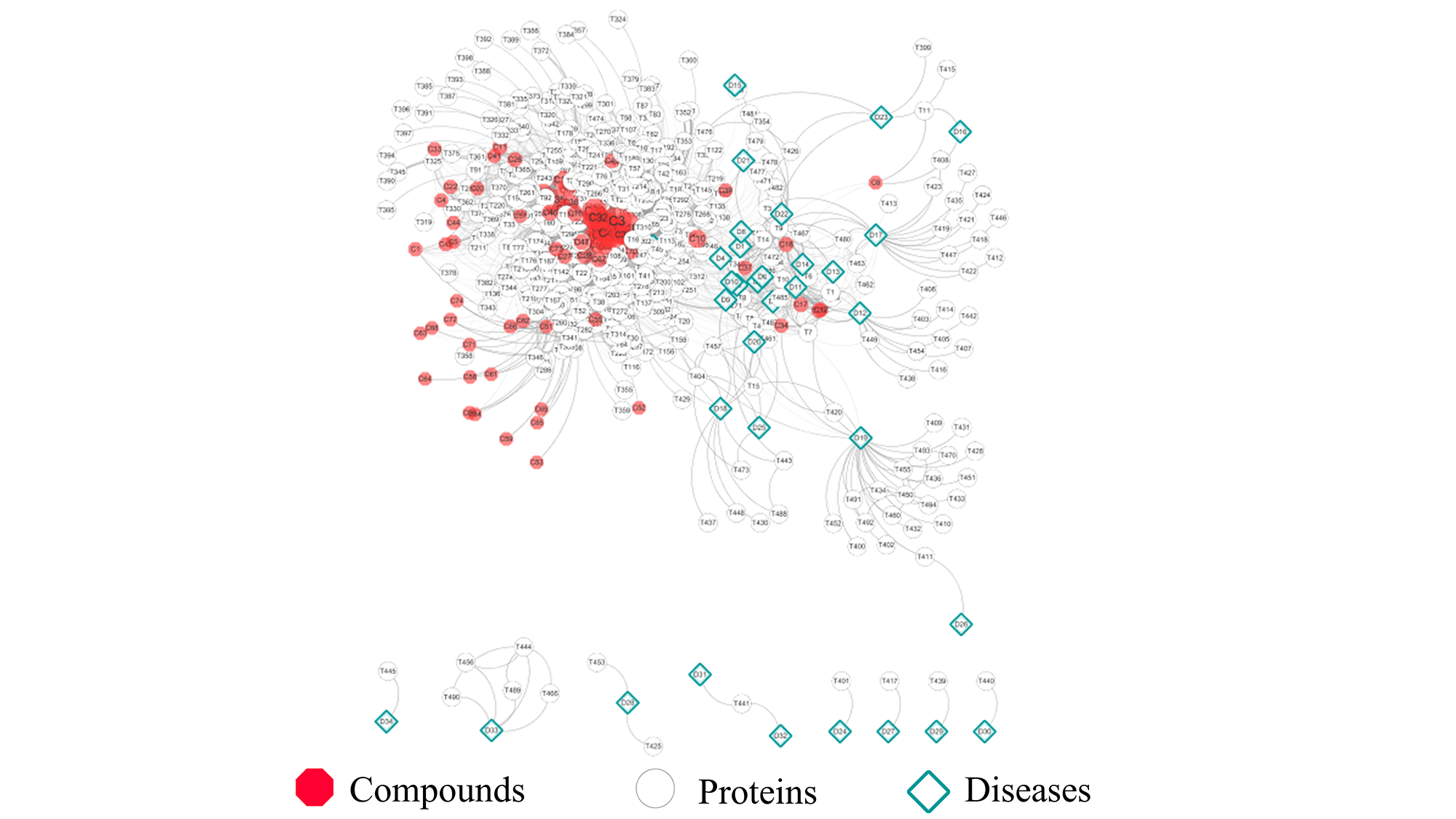


Fig. S2. The expanded compounds-targets-cardiovascular diseases interaction network of compound *Salvia miltiorrhiza*


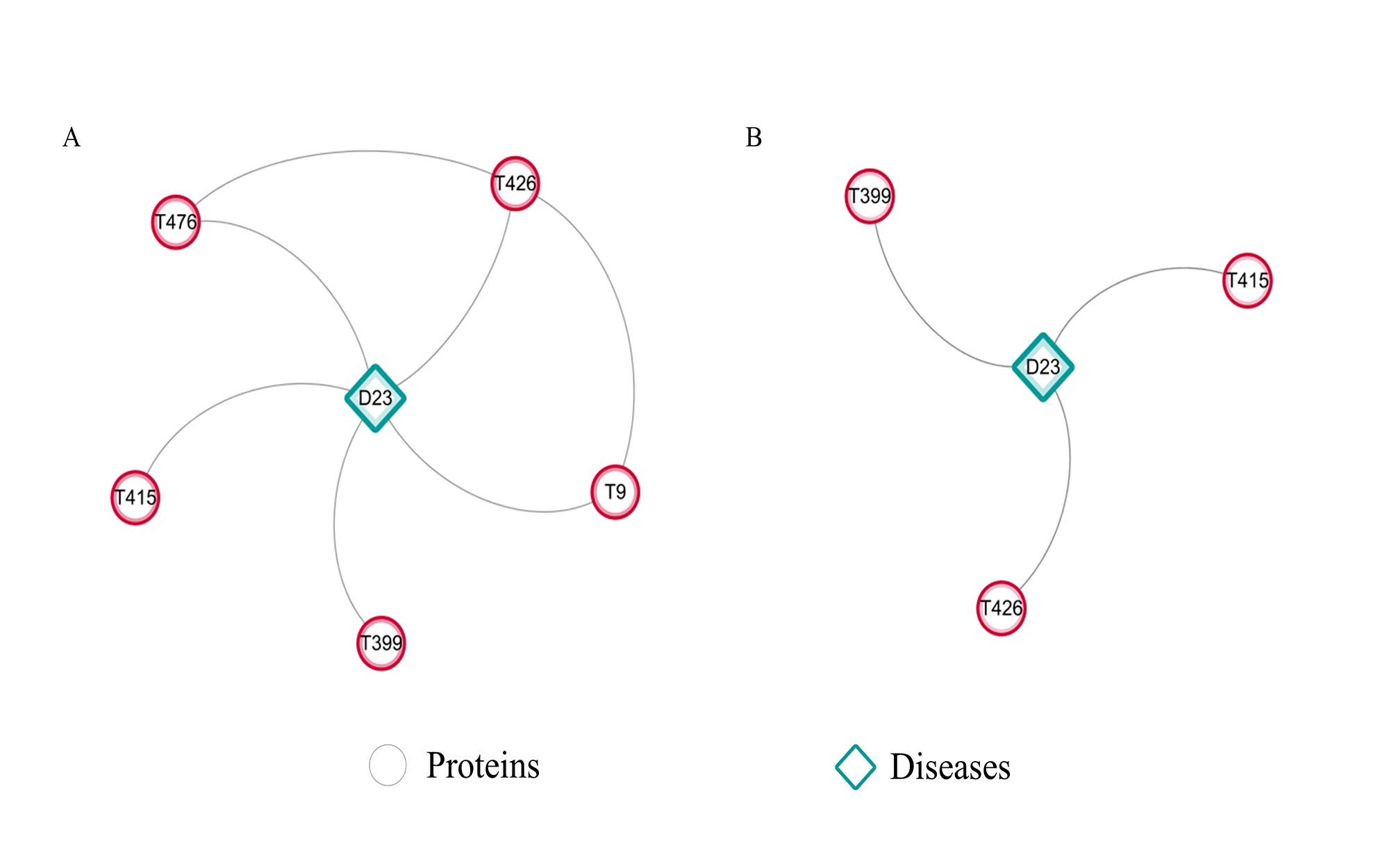


Fig. S3. D23 centered modules with path length of 1 mining from expanded (A) and original (B) network


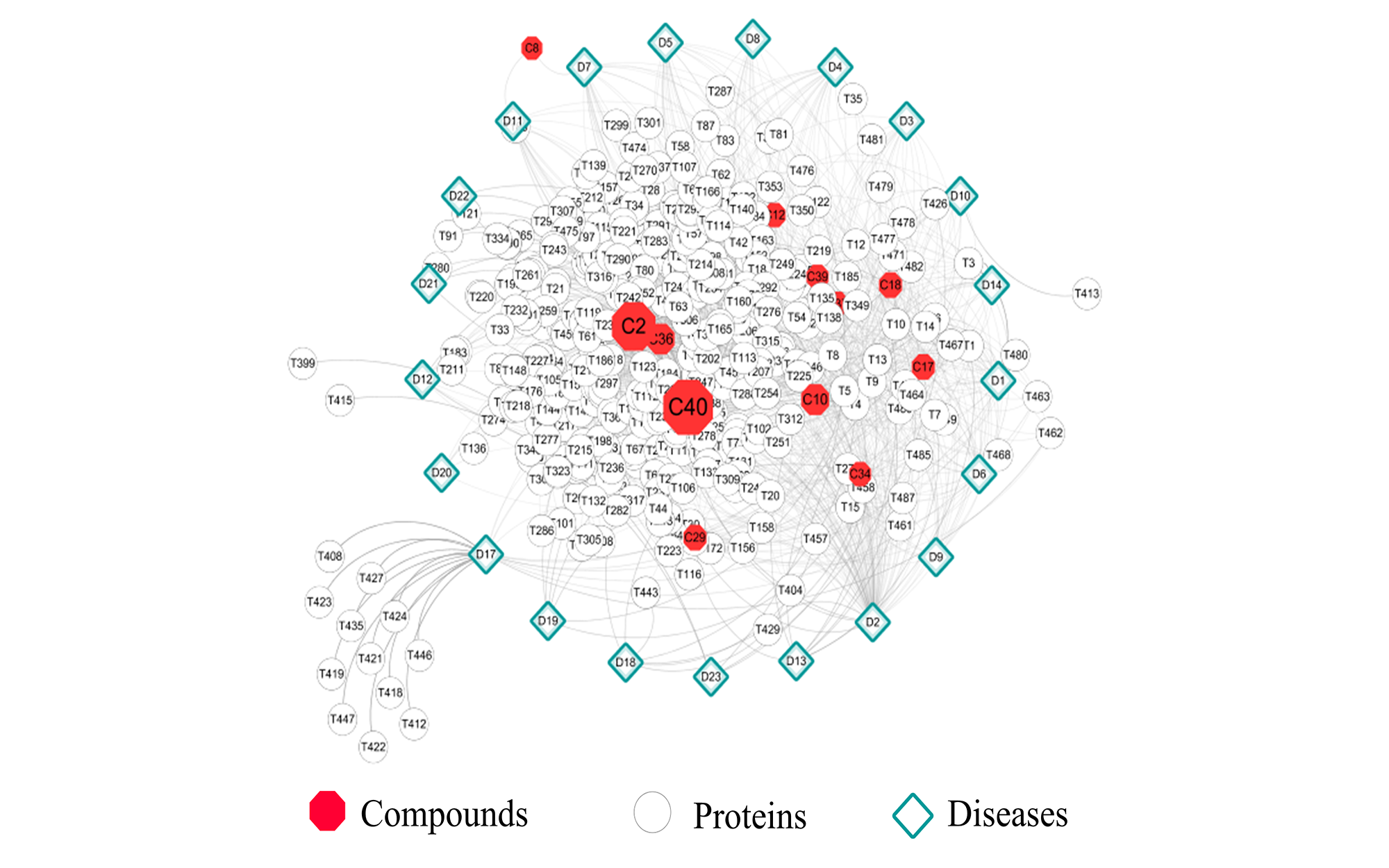


Fig. S4. D23 centered module with path length of 3 mining from expended network


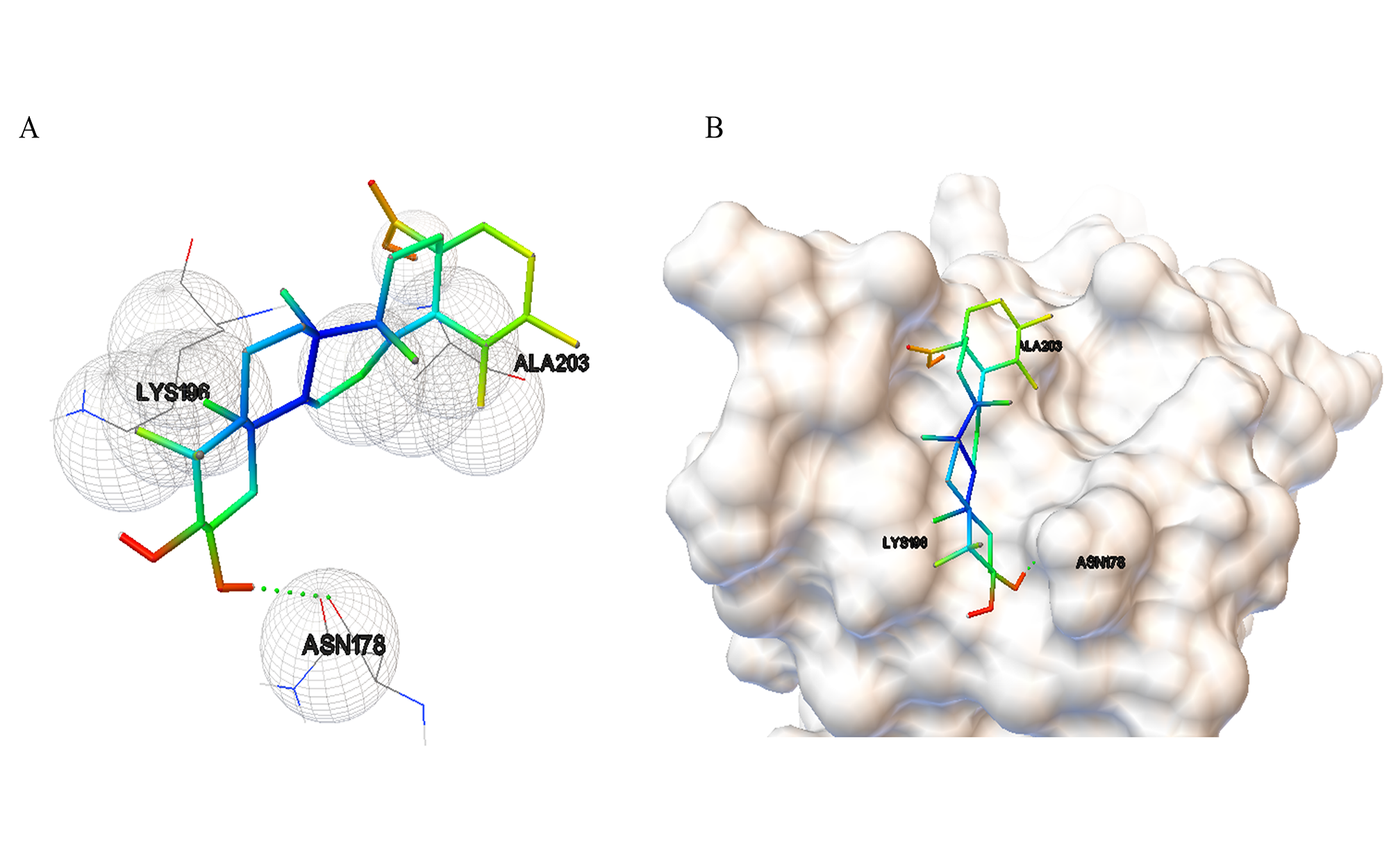


Fig. S5. Molecular docking results of 2α-Hydroxy Ursolic Acid (C2)-Insulin receptor substrate 1 (T10). PDB ID: 5U1M; Binding affinity: -6.5kcal/mol; Residues of H-Bound: ASN178.


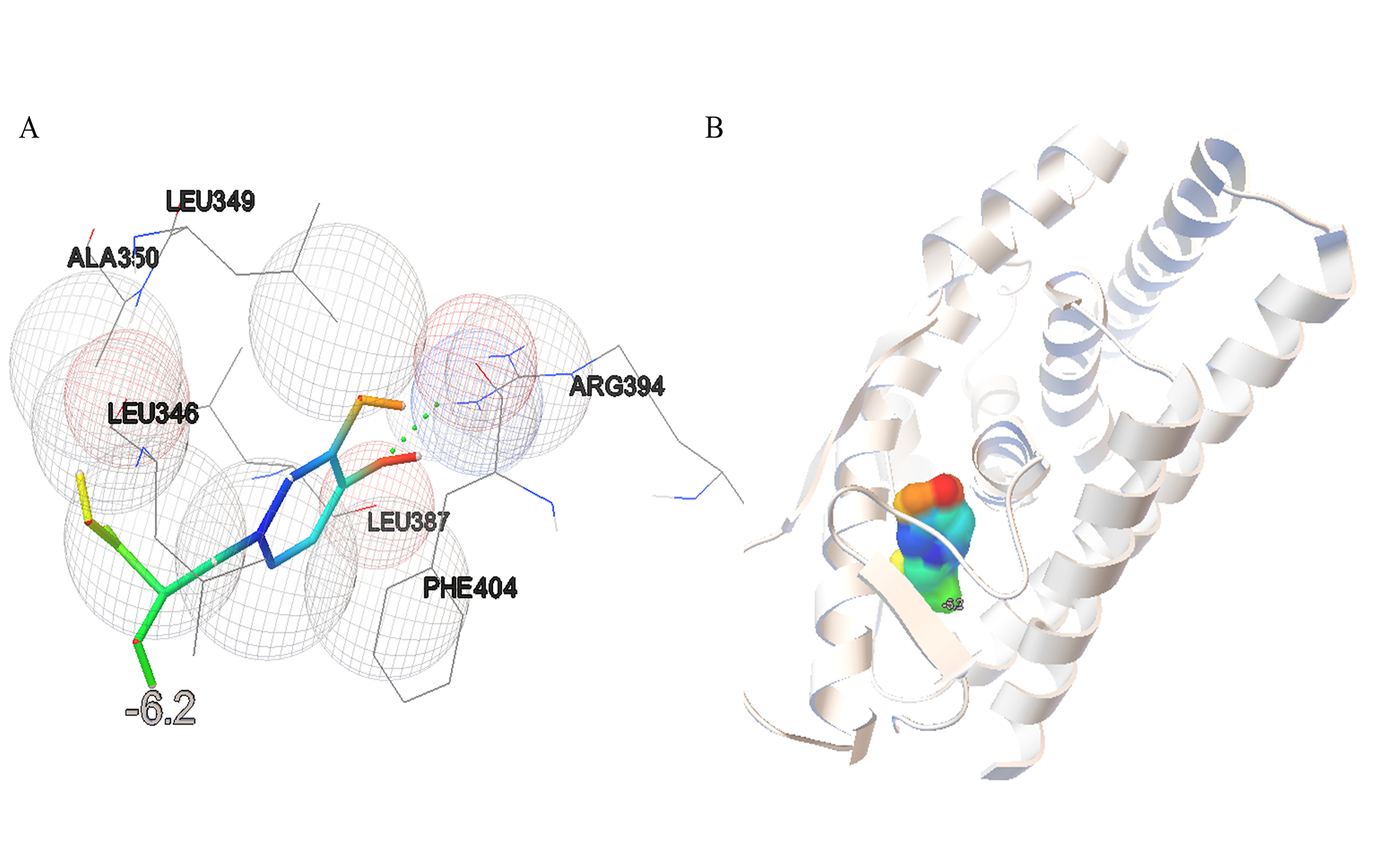


Fig. S6. Molecular docking results of Danshengsu (C12)-Estrogen receptor (T486). PDB ID: 3OS8; Binding affinity: -6.2kcal/mol; Residues of H-Bound: ARG394.


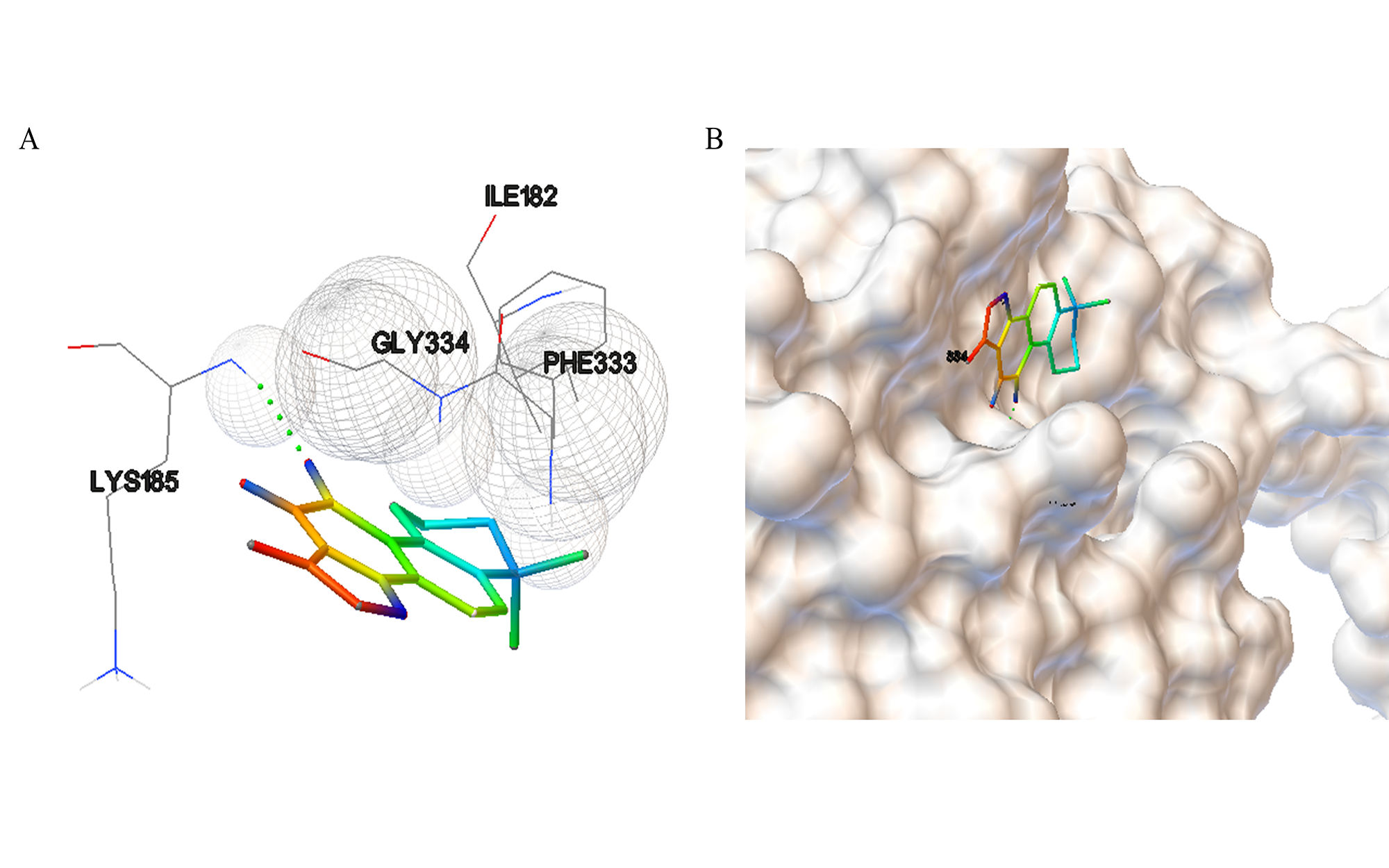


Fig. S7. Molecular docking results of Tanshinone IIA (C40)-ATP-sensitive inward rectifier potassium channel 11 (T4). PDB ID: 6C3O; Binding affinity:-7.7kcal/mol; Residues of H-Bound: LYS185.


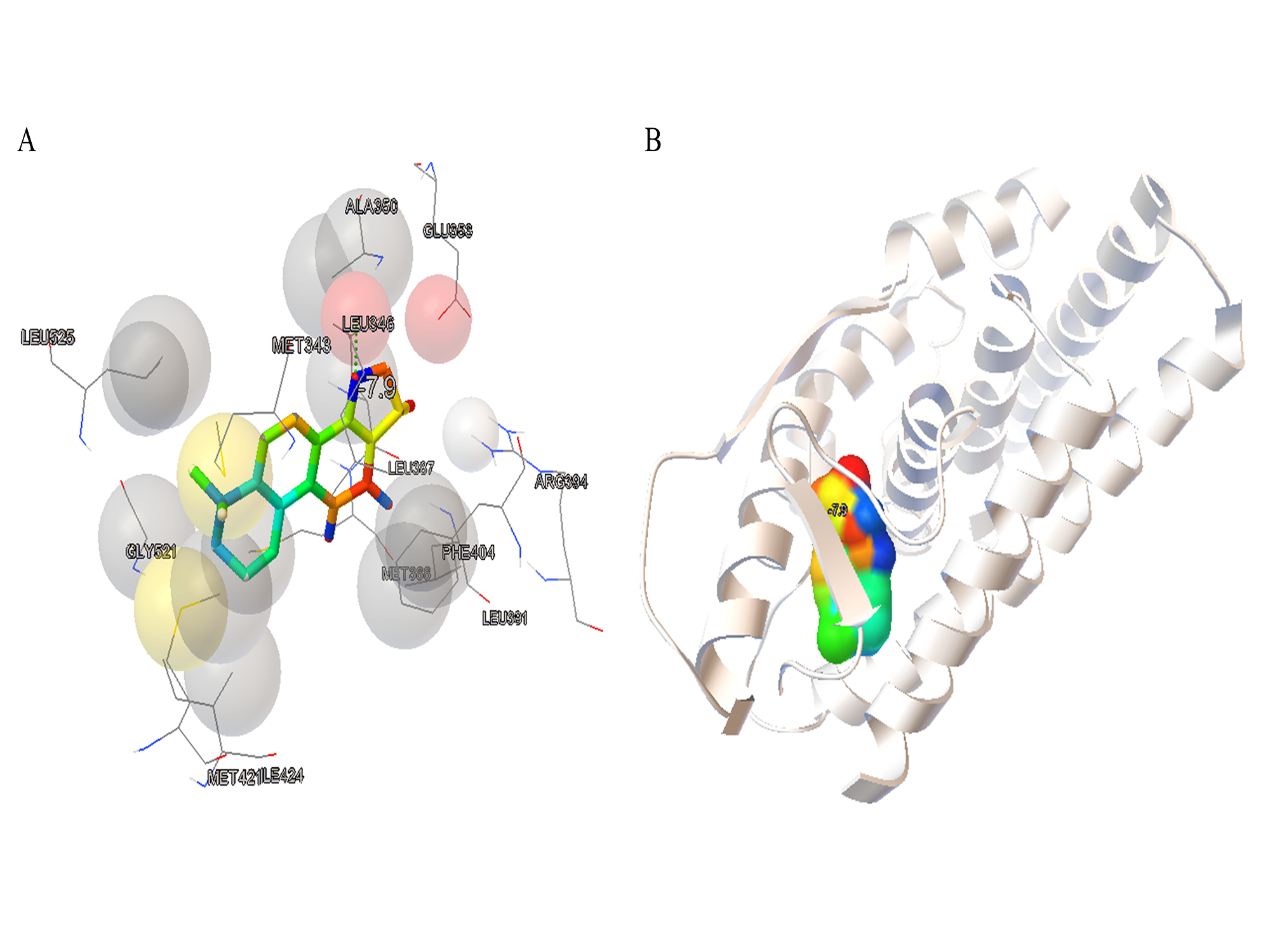


Fig. S8. Molecular docking results of Cryptotanshinone (C10)-Estrogen receptor (T486). PDB ID: 3OS8; Binding affinity: -7.9kcal/mol; Residues of H-Bound: LEU346.
